# Carcinome verruqeux de la verge

**DOI:** 10.11604/pamj.2016.24.188.9788

**Published:** 2016-07-01

**Authors:** Ali Beddouche, Adil Kallat

**Affiliations:** 1 Service d’Urologie A, Hôpital Ibn Sina, CHU Rabat, Maroc

**Keywords:** Carcinome, verruqueux, verge, pénectomie, Carcinoma, verrucous, penis, penectomy

## Image en médecine

Mr AB, âgé de 44 ans, sans antécédents particuliers. Il avait consulté pour des néoformations de la verge évoluant depuis 1 an, accompagné de signes urinaires à type de dysurie et pollakiurie, dans un contexte d’apyrexie et de conservation de l’état général. L’examen des organes génitaux externes mettait en évidence 3 tumeurs fixes, exophytiques, rouges, en “chou-fleur”, à centre blanchâtre ulcéré. L’IRM pelvienne montrait une importante infiltration des deux corps caverneux et de l’urètre. Les biopsies ont mis en évidence une tumeur bien différenciée avec prolifération épithéliale papillomateuse et hyperkératose, sans anomalies cytonucléaires. Le patient a subi une pénectomie totale avec dérivation urinaire par urétrostomie périnéale. L’examen anatomopathologique de la pièce opératoire a confirmé le diagnostic de carcinome verruqueux de la verge. L’évolution était favorable, avec un recul de 18 mois, sans récidive locale.

**Figure 1:**
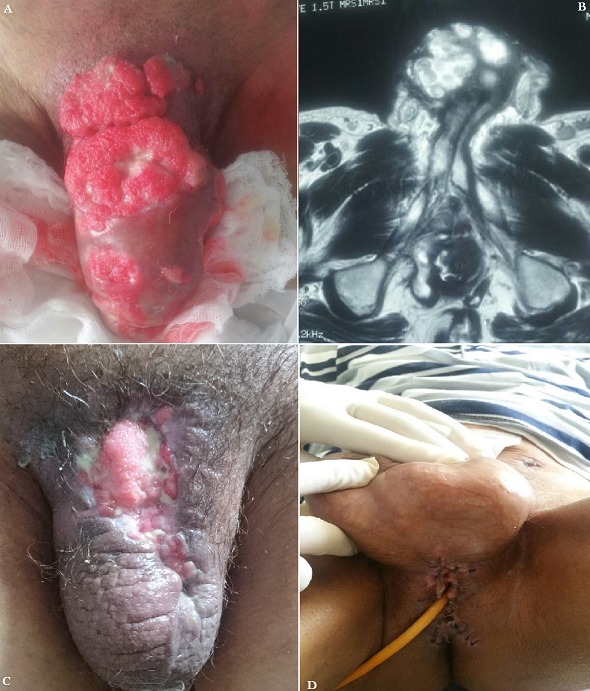
A) tumeurs en “chou-fleur”; B) IRM de la verge, infiltration des corps caverneux et de l’urètre; C) pénectomie totale; D) urétrostomie cutanée

